# Peripheral Nerve Focused Ultrasound Lesioning—Visualization and Assessment Using Diffusion Weighted Imaging

**DOI:** 10.3389/fneur.2021.673060

**Published:** 2021-07-09

**Authors:** Matthew R. Walker, Jidan Zhong, Adam C. Waspe, Karolina Piorkowska, Lananh N. Nguyen, Dimitri J. Anastakis, James M. Drake, Mojgan Hodaie

**Affiliations:** ^1^Institute of Medical Science, University of Toronto, Toronto, ON, Canada; ^2^Division of Brain, Imaging & Behaviour, Krembil Research Institute, University Health Network, Toronto, ON, Canada; ^3^Centre for Image Guided Innovation and Therapeutic Intervention, Hospital for Sick Children, Toronto, ON, Canada; ^4^Department of Medical Imaging, University of Toronto, Toronto, ON, Canada; ^5^Laboratory Medicine Program, University Health Network and University of Toronto, Toronto, ON, Canada; ^6^Department of Surgery, Toronto Western Hospital, University Health Network and University of Toronto, Toronto, ON, Canada; ^7^Department of Neurosurgery, Hospital for Sick Children, Toronto, ON, Canada; ^8^Department of Neurosurgery, Toronto Western Hospital, University Health Network, Toronto, ON, Canada

**Keywords:** magnetic resonance-guided focused ultrasound, high intensity focused ultrasound, diffusion weighted imaging, diffusion tensor imaging, tractography, peripheral nerves, neuromodulation

## Abstract

**Objectives:** Magnetic resonance-guided focused ultrasound (MRgFUS) is a non-invasive targeted tissue ablation technique that can be applied to the nervous system. Diffusion weighted imaging (DWI) can visualize and evaluate nervous system microstructure. Tractography algorithms can reconstruct fiber bundles which can be used for treatment navigation and diffusion tensor imaging (DTI) metrics permit the quantitative assessment of nerve microstructure *in vivo*. There is a need for imaging tools to aid in the visualization and quantitative assessment of treatment-related nerve changes in MRgFUS. We present a method of peripheral nerve tract reconstruction and use DTI metrics to evaluate the MRgFUS treatment effect.

**Materials and Methods:** MRgFUS was applied bilaterally to the sciatic nerves in 6 piglets (12 nerves total). T1-weighted and diffusion images were acquired before and after treatment. Tensor-based and constrained spherical deconvolution (CSD) tractography algorithms were used to reconstruct the nerves. DTI metrics of fractional anisotropy (FA), and mean (MD), axial (AD), and radial diffusivities (RD) were measured to assess acute (<1–2 h) treatment effects. Temperature was measured *in vivo* via MR thermometry. Histological data was collected for lesion assessment.

**Results:** The sciatic nerves were successfully reconstructed in all subjects. Tract disruption was observed after treatment using both CSD and tensor models. DTI metrics in the targeted nerve segments showed significantly decreased FA and increased MD, AD, and RD. Transducer output power was positively correlated with lesion volume and temperature and negatively correlated with MD, AD, and RD. No correlations were observed between FA and other measured parameters.

**Conclusions:** DWI and tractography are effective tools for visualizing peripheral nerve segments for targeting in non-invasive surgical methods and for assessing the microstructural changes that occur following MRgFUS treatment.

## Introduction

Magnetic resonance-guided focused ultrasound (MRgFUS) is a technique to thermally ablate targeted tissue using MR imaging for navigation (1). It is non-invasive and does not involve ionizing radiation. Current clinically approved indications include essential tremor (2), prostate cancer (3), uterine fibroids (4), and bone metastases (5). There is great potential in extending the use of MRgFUS to the peripheral nervous system for treating conditions such as spasticity and chronic pain (6), which is supported by observations of FUS effects on nerve conduction (7). Ablation techniques, of which MRgFUS is a potential alternative, have been studied in peripheral nerve-related conditions including painful stump neuromas (8), peripheral nerve sheath tumors (9), inguinal neuralgia (10), and lumbar degenerative disease (11). Ablation has also been used for cancer pain relief (12, 13) and to treat tumors with proximity to neural structures (14). An understanding of the nerve involvement in these ablation procedures is of great concern whether the nerves are to be targeted directly or to be avoided.

Diffusion weighted imaging (DWI), an imaging technique sensitive to the movement of water in tissue, has the ability to image nerve fibers and measure their microstructural characteristics *in vivo* (15). Tractography algorithms permit the reconstruction of nervous system fiber connectivity based on patterns of restricted water diffusion (16). Modeling the movement of water with tensors, called diffusion tensor imaging (DTI), includes metrics for quantitative evaluation of nerve characteristics. Fractional anisotropy (FA) reflects the directionality of diffusion and has been used as a proxy for nerve integrity (16). Mean diffusivity (MD), an average of all three orthogonal tensor indices, describes the overall magnitude of diffusion and reflects the degree of water diffusion restriction within tissues regardless of fiber orientation (17). Radial diffusivity (RD), a measure of diffusion perpendicular to the primary diffusion direction, is associated with degree of myelination (18). Axial diffusivity (AD), a measure of diffusion parallel to the primary diffusion direction, is sensitive to axonal integrity (18). These DTI metrics can be used as quantitative tools to assess peripheral nerve pathology and measure microstructural changes following treatment.

Accurate imaging is imperative in non-invasive treatments such as MRgFUS as faulty targeting could exacerbate comorbidities or depress treatment outcomes. It is also important that the imaging characteristics are well-understood in order to properly assess treatment response and inform treatment parameters. Conventional MR sequences, such as T1- and T2-weighted imaging, are limited as they cannot selectively visualize peripheral nerves or quantify nerve integrity or injury. Peripheral nerves have been visualized using MR neurography (19) and selective excitation techniques (20), however these approaches do not provide quantitative assessment of nerve changes after treatment. Targeting by atlas or structural images only is also limited in the ability to account for subject variability and specificity in identifying tracts of interest (21). Tractography can remedy these limitations by enhancing navigation in treatments such as transcranial thalamotomy for essential tremor (22, 23) and deep brain stimulation (21, 24, 25), resulting in improved targeting accuracy and patient outcomes.

Early work in DTI suggests its potential to evaluate peripheral nerve injury and regeneration *in vivo* (26, 27). Diffusion imaging permits longitudinal assessment and, in the case of animal studies, obviates the need for large subject numbers to be sacrificed at multiple time points, providing insight into cellular changes in lieu of histological data. Imaging of peripheral nerves has associated technical challenges due to the complexity and variability of the peripheral nervous system and surrounding muscle tissue, which is also fibrous and thus carries an anisotropic diffusion signature. Previous work has shown the importance of employing appropriate diffusion processing and tractography techniques in order to achieve anatomically accurate results (24).

There is a paucity of MRgFUS lesioning studies focusing on peripheral nerves and a greater limitation on the use of DWI to guide treatment. This is primarily due to the technical limitations associated with the accurate anatomical identification of these nerves. In this study, we investigate the use of diffusion tractography for targeting the sciatic nerves of piglets in ablative MRgFUS and DTI metrics for assessing the microstructural changes following treatment. Histological lesion analysis provides insight into cellular changes after treatment and their correlations with imaging and treatment parameters.

## Materials and Methods

### Animal Model

These experiments were approved by the Animal Care Committee and Laboratory Animal Services at the Hospital for Sick Children in Toronto, Ontario, Canada. This study conforms to the policies of the Canadian Council on Animal Care (CCAC).

Six male Yorkshire piglets (average weight 6.7 ± 1.3 kg, age 24 ± 4 days) were used. The animals were pre-anesthetized with ketamine solution [10 mg/kg] (Ketalean, CDMV Inc., Quebec, Canada) intramuscularly before being intubated and anesthesia maintained with 2.5% isoflurane and 2 L oxygen via MR-compatible ventilator. Hair was removed from both thighs of the animal via shaving and commercial depilatory cream to facilitate skin surface coupling with the MRgFUS system, aided by degassed ultrasound gel.

Once prepared, the animals were transported to the MR facilities for pre-treatment imaging on a standard clinical diagnostic table. Heart rate, peripheral capillary oxygen saturation, and body temperature were monitored throughout the experiment. A circulating water blanket was used to help maintain the piglet's core body temperature around 37°C. Upon completion of the experiment, the animals were euthanized while under anesthesia via intravenous injection of sodium pentobarbital [120 mg/kg] (Euthanyl, CDMV Inc., Quebec, Canada).

### MR Imaging

DWI and T1-weighted images were acquired before and after treatment using a clinical Philips Achieva 3T MR scanner (Philips Healthcare, Best, Netherlands) and a 32-channel receive-only cardiac coil on a standard diagnostic table. For both imaging and treatment, the animals were placed in lateral decubitus position with thighs perpendicular to the long axis of the body and the leg of interest closest to the tabletop. Separate data sets were acquired for each leg with the lateromedial coverage extending from the outer skin surface to the contralateral spinal nerve roots. This position provided stability to limit potential movement of the animal and a clear path for the ultrasound beam to target the sciatic nerve.

Anatomical images were acquired with a three-dimensional T1 magnetization prepared rapid gradient echo (MPRAGE) sequence. Acquisition parameters included: repetition time (TR) 8.1 ms; echo time (TE) 3.7 ms; flip angle 8°; matrix 224 × 224; field of view (FOV) 224 × 224 mm; slice thickness 1 mm; slice number 70; voxel resolution 1 × 1 × 1 mm; number of signal averages (NSA) 4; SENSE reduction factor 2; acquisition time 14 min 35 s.

Diffusion images were collected with a SENSE-single shot spin-echo echo-planar-imaging (SE-EPI) sequence with a *b*-value of 800 s/mm^2^ and 128 diffusion encoding directions. Two additional baseline images with *b* = 0 s/mm^2^ were acquired, one each in forward and reverse phase-encode directions, for post-processing EPI-based susceptibility distortion corrections. Other diffusion scanning parameters include: TR 5845 ms; TE 106 ms; flip angle 90°; matrix 128 × 128; FOV 205 × 205 mm; slice thickness 1.6 mm; slice number 38; voxel resolution 1.6 × 1.6 × 1.6 mm; NSA 2; SENSE reduction factor 2; diffusion gradient pulse duration/time interval 15.7/52.9 ms; acquisition time 29 min 33 s.

### Image Post-processing and Tractography

Post-processing was carried out with the FSL software library (Analysis Group, FMRIB, Oxford, UK: https://fsl.fmrib.ox.ac.uk/fsl/fslwiki) (28). Corrections were performed to remedy distortions caused by EPI and susceptibility-induced off-resonance fields using the two baseline images with opposing phase-encoding as implemented in FSL “topup” (29, 30). Susceptibility-corrected images were further processed to remove distortions associated with bulk motion and eddy currents in FSL “eddy” (31). Water diffusion was modeled from fully corrected data by fitting tensors to each image voxel (32). Tensors were used to calculate scalar maps of FA, MD, AD, and RD (33). Structural and diffusion images were linearly co-registered using FSL FLIRT and manual adjustment (34). Pre- and post-treatment images within each subject were co-registered with the same method. Image processing time required was ~40 min for each subject using a moderately powerful workstation with dedicated graphical processing unit.

Fiber tracking was performed with MRtrix (Brain Research Institute, Melbourne, Australia: http://www.brain.org.au/software) (35). The response function for a single fiber population was estimated using the default threshold of FA > 0.2 (36). This response function was then used with a basis of constrained spherical deconvolution (CSD) to estimate the fiber orientation distribution (FOD) (37). CSD has been shown to be an effective tractography method in regions of complex fiber orientations and crossing fibers (38). A deterministic tractography algorithm, “SD-Stream,” was used to generate tracks (35). Tracking seeds were delineated manually at the lumbar nerve roots based on structural T1 and FOD maps. Tracts were segmented by placing inclusion regions of interest (ROI) in terminating muscle regions.

DTI metric assessment was performed by manual placement of a 2 × 2 × 1 voxel (3.2 × 3.2 × 1.6 mm^3^) ROI on the sciatic nerve overlapped by the post-treatment lesion as identified by tractography and T1 data. Measurements of FA, AD, RD, and MD were pulled from co-registered pre- and post-treatment images using the same ROI mask. Visually distinct lesion zones were manually identified and measured on T1, aided by voxel intensity thresholding, as a region of hyperintensity (zone I) concentrically surrounded by hypointensity (zone II).

### MRgFUS Treatment

Treatment was performed with a clinical MRgFUS system (Sonalleve V1, Profound Medical, Toronto, Canada). Animals were positioned on the treatment table housing the ultrasound transducer. The same lateral decubitus position was used in pre- and post-treatment imaging. Degassed ultrasound gel was applied to the skin surface with a 20 mm gel pad (Aquaflex, Parker Laboratories, New Jersey, USA) placed between the animal and treatment table to facilitate acoustic coupling with the transducer.

Imaging during treatment was accomplished using one element of the cardiac coil (16 channels) placed on top of the animal. Skin bubble images were acquired using a three-dimensional spoiled gradient echo (FFE) sequence to confirm that no air bubbles were present which may interfere with acoustic beam propagation. T1-weighted FFE images were acquired for immediate target identification and treatment cell placement using the Sonalleve planning software.

**Figure 1:**
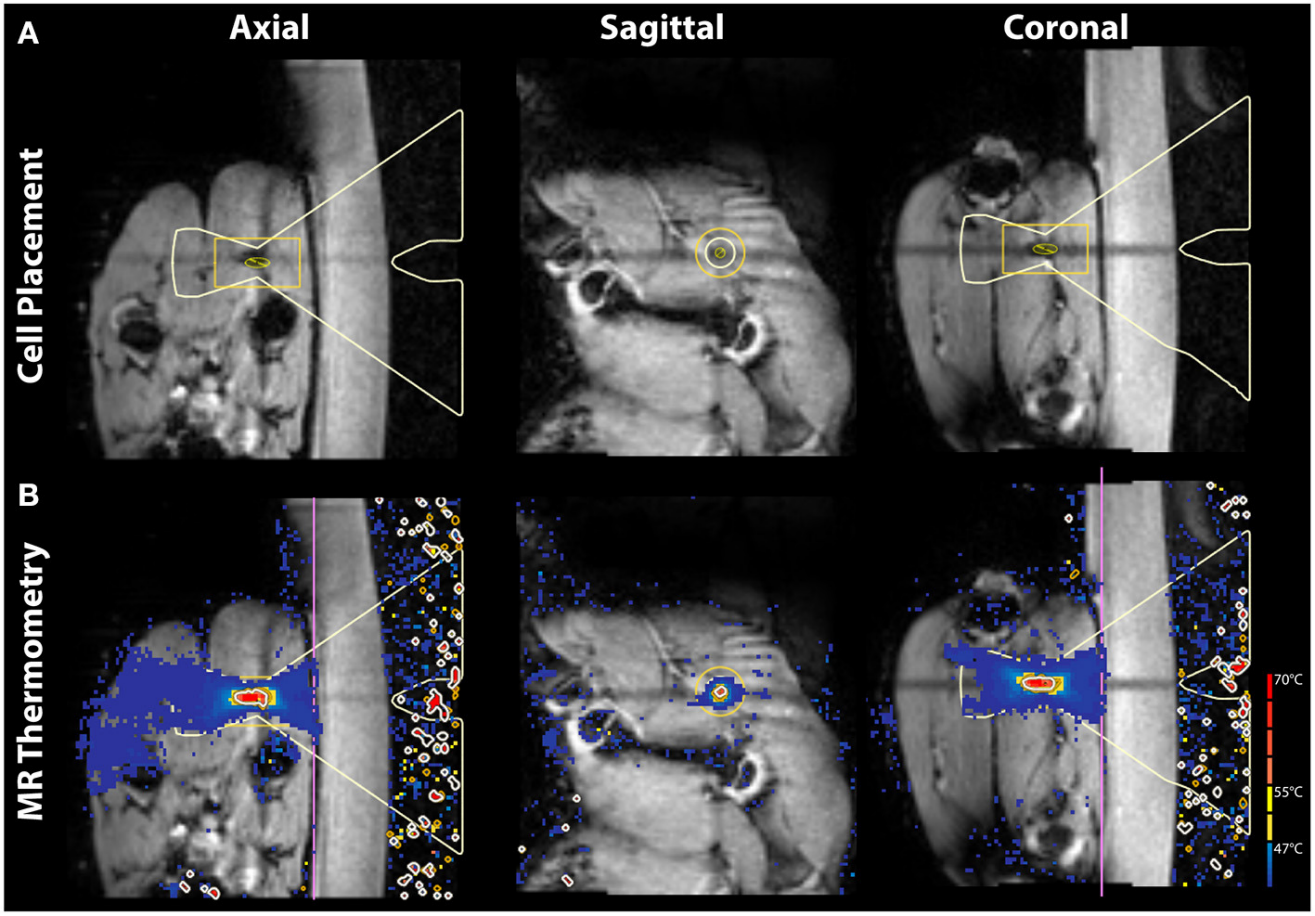
Pre-treatment planning images and in-treatment MR thermometry in axial, sagittal, and coronal view. **(A)** T1-weighted images are shown with the MRgFUS treatment cell placed on the sciatic nerve. **(B)** *In vivo* MR thermometry displaying heat deposition during treatment.

Cells were placed on the sciatic nerve, posterior to the proximal head of the adjacent femur, with guidance from pre-treatment tractography (Figure 1A). The sciatic nerves in these piglets are ~3 mm wide therefore treatment cell diameters of 4 and 8 mm were chosen to cover the whole nerve. Sonication times are fixed based on cell diameter leading to 20 and 27 s treatments for these respective cell sizes. The distance along the beam path from skin surface to treatment cell center was measured. A single sonication was used for each treatment in order to isolate the effect of a given cell size and transducer output power. All ultrasound treatments were delivered with a frequency of 1.2 MHz. Before each therapeutic exposure, one to two test sonications at reduced power of 10 W and 20 s were performed for calibration of beam focus location. Temperature was measured simultaneously with sonication via MR thermometry (Figure 1B). Specific treatment parameters for each nerve can be found in Table 1. Immediately following treatment in both legs, the animal was repositioned on the diagnostic table for post-treatment imaging (1–2 h after sonication). Identical scanning sequences were used as in pre-treatment imaging.

**Table 1:** MR-guided focused ultrasound treatment parameters.

**Animal #**	**Target**	**Treatment cell**	**Sonication**	**Input power (W)**	**Energy (J)**	**Maximum**	**Temperature**	**Zone I**	**Zone II**	**Total**
	**leg**	**diameter (mm)**	**time (s)**			**temperature (^**°**^C)**	**difference (^**°**^C)**	**volume (mm^**3**^)**	**volume (mm^**3**^)**	**volume (mm^**3**^)**
1	Left	8	27	70	1,890	57.6	18.2	41	140	181
1	Right	8	27	50	1,350	58.7	19.3	53	110	163
2	Left	8	27	110	2,970	82.2	45.7	131	564	695
2	Right	8	27	90	2,430	66.2	29.7	93	340	433
3	Left	8	27	60	1,620	53.2	20.2	59	211	270
3	Right	4	20	80	1,600	56.4	26.4	20	81	101
4	Left	4	20	90	1,800	66.9	35.6	130	410	540
4	Right	8	27	100	2,700	64.2	33.6	274	290	564
5	Left	4	20	100	2,000	65.8	32.4	326	729	1,055
5	Right	4	20	110	2,200	77.6	44.6	392	948	1,340
6	Left	4	20	120	2,400	74.9	39.3	531	809	1,340
6	Right	4	20	130	2,600	112.7	77.3	430	1,270	1,700

### Histology

Animals were euthanized immediately after post-treatment imaging (<2 h following treatment). The treated area was identified by reflecting the biceps femoris muscle to expose the sciatic nerve and lesion on surrounding muscle tissue. The treated portion of the sciatic nerve and adjacent sections of biceps femoris and semitendinosus muscles were collected. Treated muscle specimens were sampled superficially, deep or mirror to the treated area. Nerve specimens were sampled longitudinally along the axis of the beam path. Internal control specimens of both nerve and muscle were sampled several centimeters away from treated areas, confirmed to free from temperature changes via MR thermometry maps and tissue damage via gross inspection. Samples were immersion fixed with 10% neutral buffered formalin and cooled in a 4°C refrigerator for 48 h before routine histological processing. The tissue was sectioned at a 5-micron thickness at 200-micron levels and stained with hematoxylin and eosin (H&E) and luxol fast blue (LFB). Control samples were exposed to the same conditions and processing as for treated samples except for MRgFUS ablation. Thus, any differences observed between treated and control tissue would be related to MRgFUS treatment. All specimens were evaluated by a neuropathologist who was blinded to the treatment conditions of each specimen.

### Statistical Analysis

DTI metric comparisons from pre- and post-treatment lesion ROIs were carried out using paired, two-tailed *t*-tests. Significance was taken as *P* < 0.05. Pearson correlations were performed between maximum temperature, temperature change from baseline, lesion volumes (zones I and II), DTI metrics, and output power (absolute and normalized by skin-to-focus distance). Statistical analysis was performed using SPSS version 23 (IBM, Inc.).

## Results

### Peripheral Nerve Imaging

**Figure 2:**
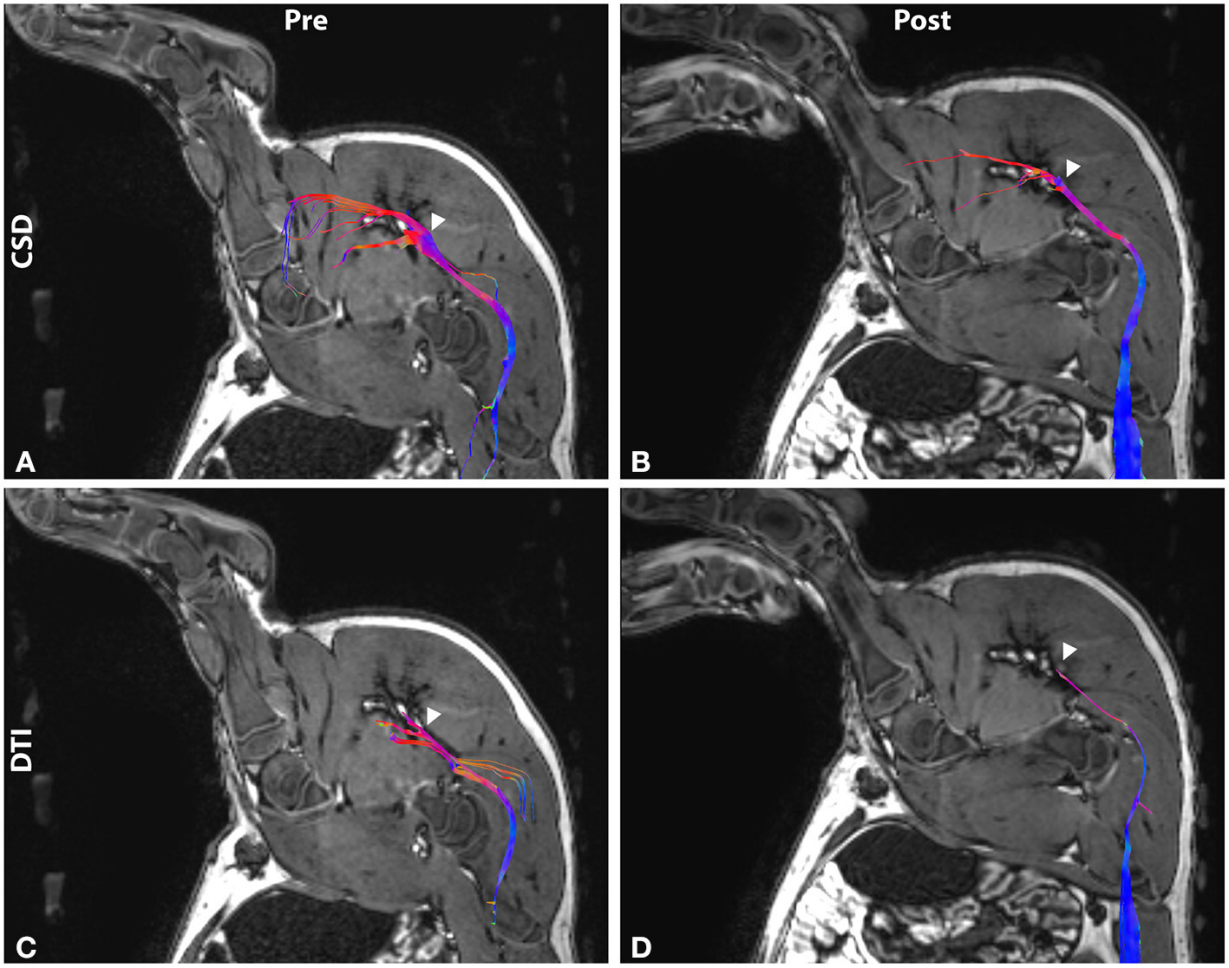
Tractography before and after MRgFUS treatment in sagittal view. Tracts are reconstructed using **(A,B)** constrained spherical deconvolution (CSD) and **(C,D)** diffusion tensor imaging (DTI) methods. Tracts are overlaid on T1-weighted images and colored by orientation: anterior-posterior (blue); superior-inferior (red); left-right (green). Arrowhead indicates treatment target.

On T1-weighted imaging, the sciatic nerve presented as a moderate hyperintensity associated with suppressed signal from nearby blood vessels. Surrounding fat and connective tissue of similar signal intensity to the nerve introduced uncertainty in assessing nerve position at all points in its caudal trajectory from the lumbar plexus. However, the sciatic nerves were successfully reconstructed via both single tensor and CSD tractography in all subjects (Figure 2). Tract models were observed extending from the vertebral roots, through the lumbar plexus, and terminating in the muscles of the leg posterior to the femur.

### MRgFUS Treatment Assessment

Tract abnormalities following MRgFUS treatment were observed in both single tensor and CSD reconstruction models (Figure 2). For the tensor model, tracts were discontinuous within the lesion in 8 of the 12 nerves imaged. DTI tracts that did extend into the lesion displayed narrowing and decreased fiber density. In the CSD model, tract continuity was maintained through the lesion in all cases with narrowing and decreased fiber density within the treatment area.

**Figure 3:**
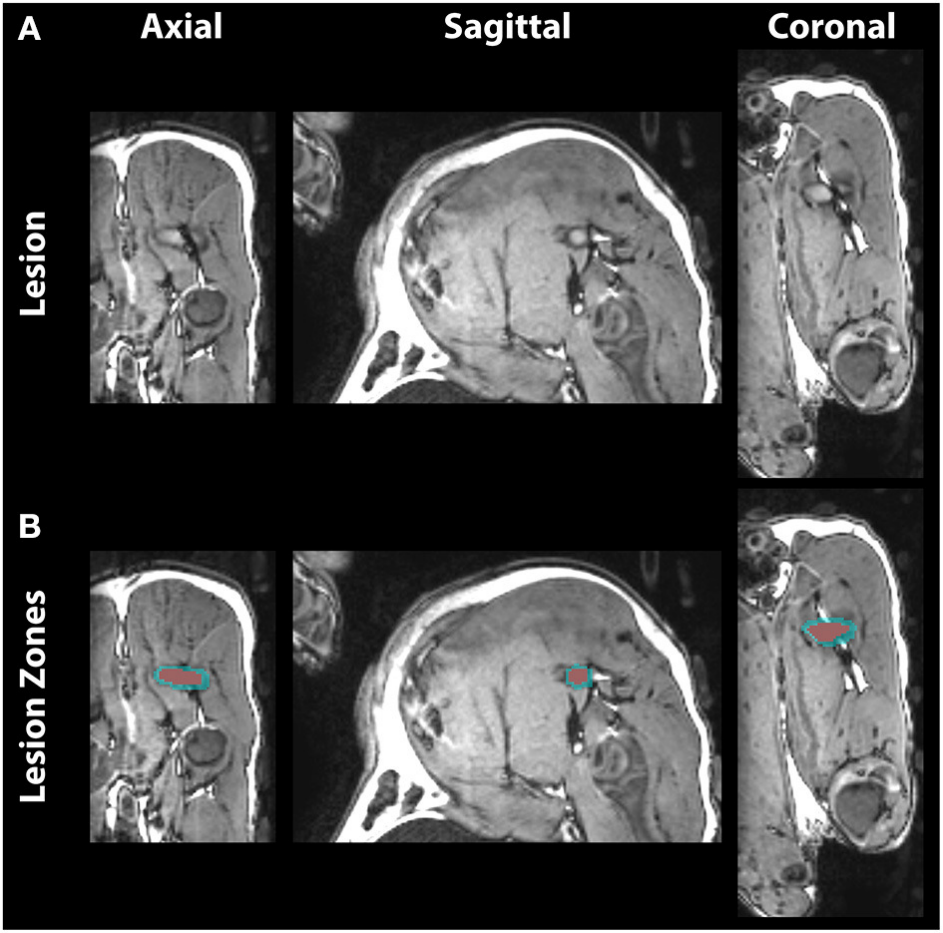
Post-treatment T1-weighted images in axial, sagittal, and coronal view. **(A)** The lesion on the sciatic nerve shows distinct zones of hyperintense necrotic core and surrounding hypointense edema. **(B)** Lesion voxels were segmented into central zone I (red) and surrounding zone II (blue).

Lesions were identified on T1 images by regions of hyperintensity (zone I) surrounded by hypointensity (zone II) (Figure 3). Zone I ranged 20–531 mm^3^ with an average of volume of 207 mm^3^. Zone II ranged 81 to 1,270 mm^3^ with an average of 492 mm^3^. Total volume ranged 101–1,700 mm^3^ with an average of 699 mm^3^. Individual volume measurements and treatment parameters are listed in Table 1.

Significant correlations were observed between output power and maximum lesion temperature (correlation coefficient *r* = 0.81, *P* = 0.001), temperature difference (*r* = 0.86, *P* = 0.0004), lesion zone I volume (*r* = 0.82, *P* = 0.001), zone II volume (*r* = 0.86, *P* = 0.0003), and total lesion volume (*r* = 0.88, *P* = 0.0001). Output energy was also correlated with maximum temperature (*r* = 0.64, *P* = 0.025), temperature difference (*r* = 0.64, *P* = 0.025).

**Figure 4:**
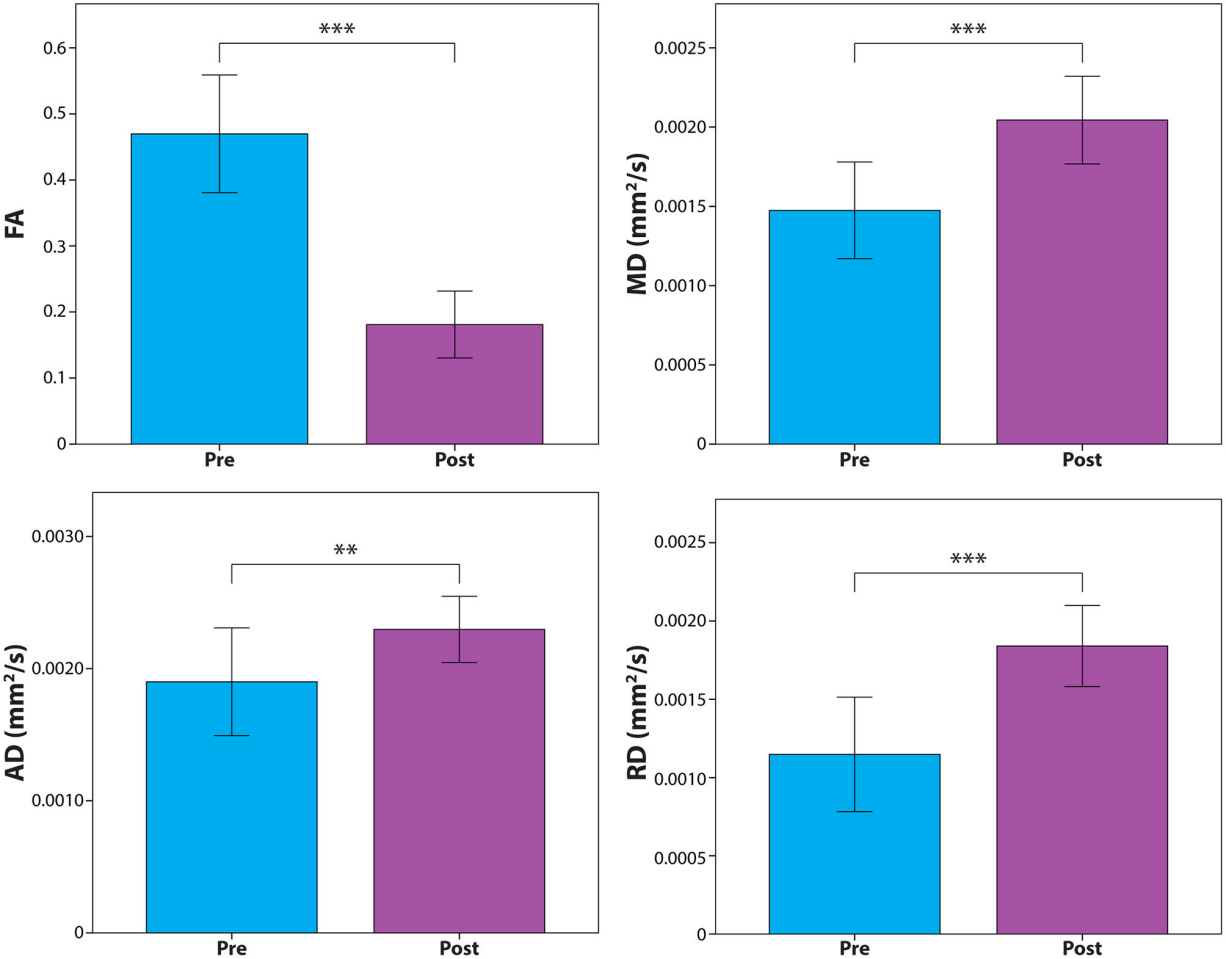
Diffusion tensor metric assessment of the treated sciatic nerve segment before and after MRgFUS. Results show significant changes in fractional anisotropy (FA) and mean (MD), axial (AD), and radial diffusivity (RD). **P < 0.01; ***P < 0.001.

DTI metrics measured over the sciatic nerve within the lesion immediately after treatment compared to the pre-treatment baseline revealed significantly decreased FA (*P* = 0.00008), and increased MD (*P* = 0.0008), AD (*P* = 0.01), and RD (*P* = 0.0001). Results are depicted graphically in Figure 4.

Significant negative correlations were also observed for output power with diffusivity changes relative to baseline: MD (*r* = −0.77, *P* = 0.004), AD (*r* = −0.70, *P* = 0.011), and RD (*r* = −0.70, *P* = 0.011). No correlations were seen between power and FA.

Maximum temperature and temperature difference were well-correlated with total lesion volume (*r* = 0.81, *P* = 0.004 and *r* = 0.80, *P* = 0.005, respectively) and zone II volume (*r* = 0.88, *P* = 0.001 and *r* = 0.89, *P* = 0.001, respectively) and relatively weakly correlated with zone I volume (*r* = 0.65, *P* = 0.023 and *r* = 0.67, *P* = 0.017, respectively). Temperature difference correlated with MD (*r* = −0.68, *P* = 0.016) and AD (*r* = −0.67, *P* = 0.016) but not significantly with RD (*r* = −0.56, *P* = 0.058) or FA (*r* = 0.05, *P* = 0.87).

**Figure 5:**
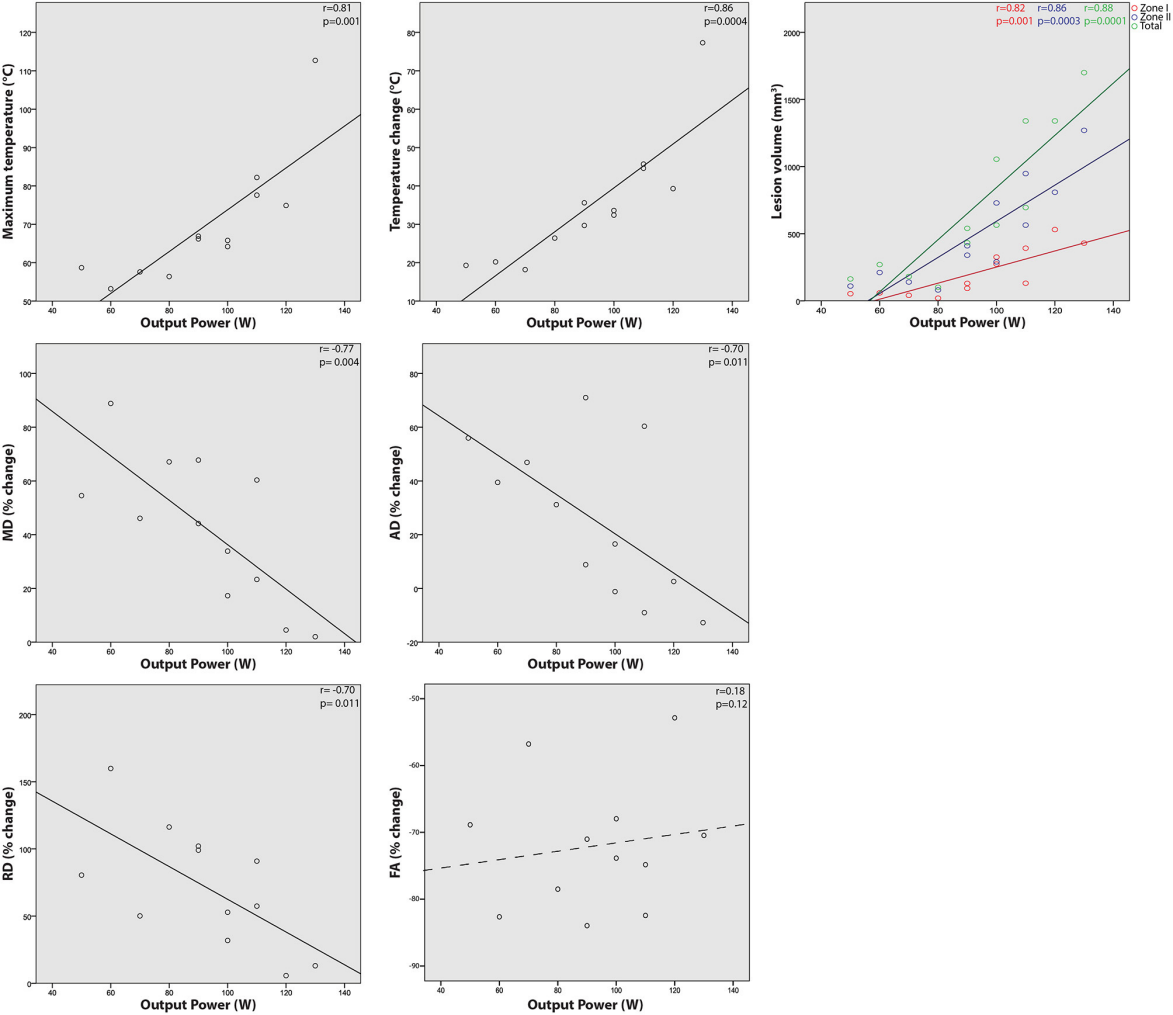
Relationships between single sonication MRgFUS output and treatment results. Correlations are shown between transducer output power and resulting maximum temperature, temperature change from baseline, percent change in fractional anisotropy (FA), mean (MD), axial (AD), and radial diffusivity (RD), and lesion volume (zone I, II, and total).

Output power normalized by skin-to-focus distance (measured as distance from skin surface to center of treatment cell) was weakly correlated with only maximum temperature (*r* = 0.58, *P* = 0.047) and temperature difference (*r* = 0.68, *P* = 0.015)—less significant than correlations with absolute output power. Selected correlations are shown graphically in Figure 5.

### Histological Analysis

**Figure 6:**
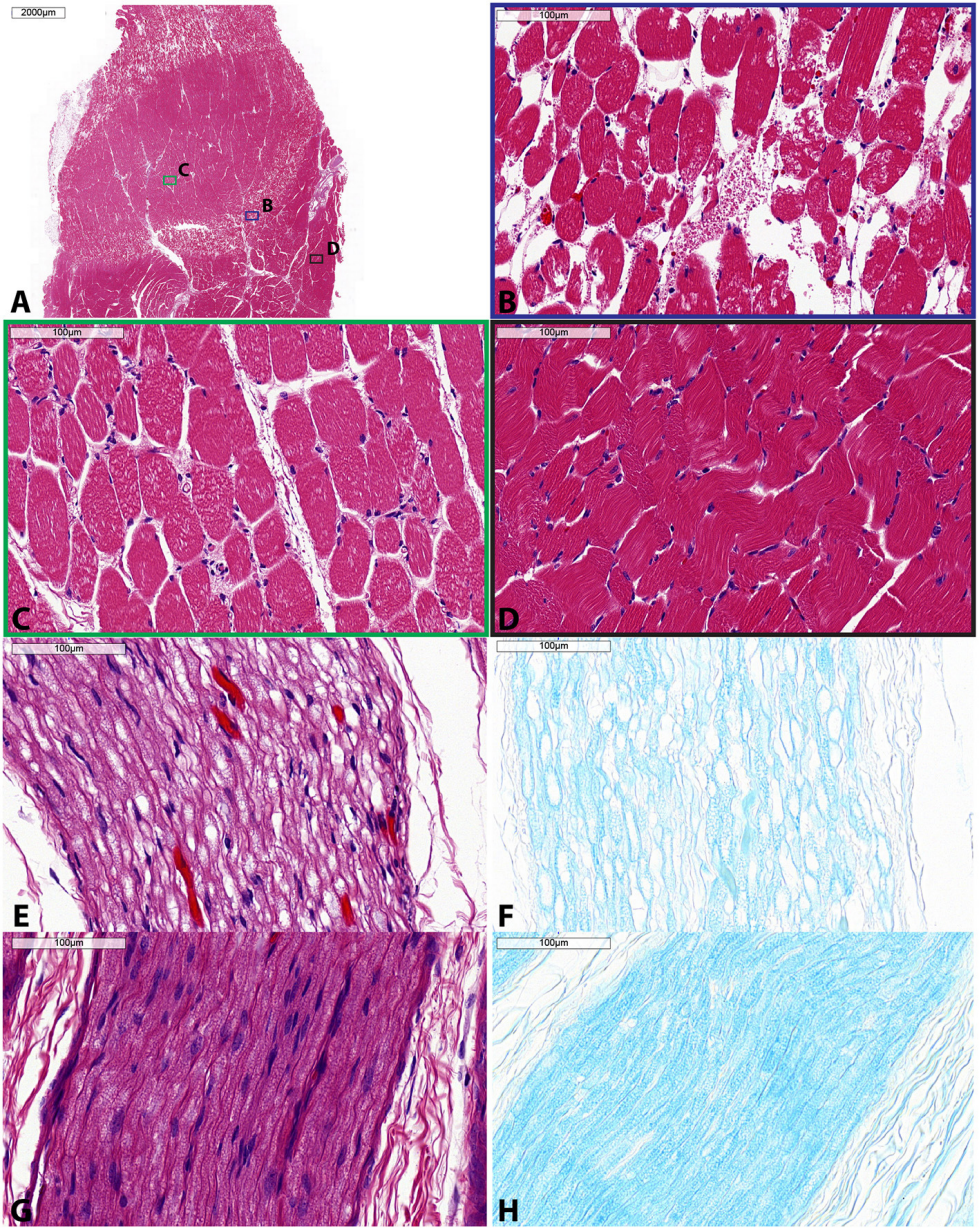
Histologic specimens taken of muscle **(A–D)** and sciatic nerve **(E–H)** following single sonication MRgFUS treatment. **(A)** Low magnification showing zonal changes within the muscle—severe, moderate, and mild. Within this muscle specimen, colored boxes indicate areas of high magnification shown in panels **(B–D)**, each corresponding to these zonal changes. **(B)** High magnification of severe muscle involvement (blue) shows diffuse edema, myofiber vacuolation, and myofiber dropout. **(C)** High magnification of moderate muscle involvement (green) shows perimysial and endomysial edema, myofiber vacuolation, and myofiber necrosis. **(D)** High magnification of mild muscle involvement (black) shows mild myofiber atrophy and endomysial edema. **(E)** High magnification of involved nerve shows endoneurial edema, demyelination, and axonal fragmentation as highlighted by **(F)** LFB stain. **(G)** High magnification of control nerve H&E stain and **(H)** LFB stain. H&E, hematoxylin and eosin; LFB, luxol fast blue. Scale bar is 2,000 μm in **(A)** and 100 μm for **(B–H)**.

Gross examination of the dissected tissue revealed pallor of the surrounding muscles and red discoloration of the perineural tissue, further confirming accurate and sufficient delivery of the acoustic energy to the sciatic nerve. Control specimens appeared unremarkable (i.e., no signs tissue damage observed). Microscopic examination of the muscle specimens showed pathologic changes in all areas sampled, superficially, deep or mirror, indicating sensitivity to the acoustic energy regardless of location. All specimens showed changes in a zonal or gradient pattern. Figures 6A–D shows a representative zonal pattern where on one end, minimal endomysial edema was present while on the other end, extensive edema and myofiber dropout were present. Nerve specimens (Figures 6E–H) showed marked changes. On H&E, specimens showed extensive perineurial and endoneurial edema. LFB stain highlighted loss of myelin while H&E/LFB dual stain highlighted axonal loss. Control specimens for muscle and nerve were unremarkable histologically.

## Discussion

In this study, we have demonstrated the feasibility of using diffusion MR tractography to identify and visually reconstruct peripheral nerves and guide their ablative treatment with a clinical MRgFUS system. Further, we have shown the ability of DTI metrics to quantitatively assess the acute changes following treatment. MRgFUS informed by tractography is capable of producing thermal lesions focused on peripheral nerves with minimal damage to surrounding tissue. DTI metrics demonstrate significant microstructural changes to nervous tissue in the form of decreased FA and increased MD, AD, and RD, the latter three of which were found to negatively correlate with transducer output power. Histological analysis verified the damage to the nerves and sharp transition zones from lesions and adjacent untreated tissue.

Experiments of constriction injury in rabbit sciatic nerves have reported microstructural alterations consistent with decreased FA and increased MD and RD (39, 40). However, we observed increased AD while these authors cited significant (40) and non-significant (39) decreases in AD. In constriction injury, it appears that the chronic compression primarily restricts water motion parallel with the nerve and secondarily drives an inflammatory response of axonal swelling and loosening of the myelin sheath. In acute MRgFUS, the thermal ablation results in axonal fragmentation and the accumulation of cell debris which, along with the influx of inflammatory fluid and loosening myelin, contributes to a change toward isotropic diffusion within the nerve. RD, which is associated with degree of fiber myelination, experienced the largest change relative to pre-treatment baseline. Thus, we suspect the myelin disruption and widening of the periaxonal space as seen on histology to be the driving factor behind the results of increased diffusivity and decreased directionality. This is supported by a previous report (7) which identified axonotmesis, where the axons and myelin sheath are damaged but the gross structure (i.e., epineurium, perineurium, and endoneurium) remains intact, in rat sciatic nerves following FUS exposure. Our tractography results corroborate this further as fiber reconstruction with CSD within the lesion was still possible after treatment. Narrowing of reconstructed fibers and decreased fiber density within the lesion is indicative of some axonal damage and decreased diffusion directionality.

In addition to significant changes in DTI metrics at the group level, negative correlations were observed for MD, AD, and RD with transducer output power. That is, with increasing power more modest alterations to diffusivities were measured. Similar correlations were seen between temperature change from baseline and MD and AD with the RD correlation failing to reach statistical significance (*P* = 0.058). FA changes did not correlate with any other observable. We interpret these FA findings as a possible ceiling effect to the FA decrease in which the nervous tissue has become sufficiently disordered such that further acoustic energy will not further affect diffusion directionality within the nerve. Negative correlations between the diffusivities and both output power and temperature were unexpected, however. We hypothesize that the damage associated with higher sonication intensity and lesion temperature results in increasing damage and accumulation of cellular debris which acts to inhibit the increased motion of water within the nerve. RD exhibited greater relative change with power than both MD and AD. Thus, RD would be the most substantially affected in this regard as myelin disruption-associated RD changes are suspected to be the driving factor behind the diffusivity increases overall.

DWI has been used previously as a monitoring tool immediately after the MRgFUS treatment of bone in *ex vivo* lamb legs (41). Correlations were reported between applied energy, maximum temperature, and lesion volume, which was expected and is confirmed in the present study. Giles et al. also observed a positive correlation between apparent diffusion coefficient (ADC) and applied energy, maximum temperature, and lesion volume in muscle tissue adjacent to treated bone at time points ranging from <1–50 min after treatment. While this result differs from the anti-correlation currently presented, several important differences exist between studies: Giles et al. measure only ADC in muscle tissue adjacent to bone in room temperature *ex vivo* subjects at time points <50 min post-treatment. Conversely, we measured multiple DTI metrics in nervous tissue *in vivo* 1–2 h after sonication. The differences in acoustic energy attenuation between bone and soft tissue (42), thermal diffusion dynamics due to blood flow (43), and differing time points complicates direct comparison of the two studies. Further investigation is needed regarding the time evolution of MRgFUS lesions and microstructural dynamics of nerve and muscle tissue.

DTI metrics have been used longitudinally to evaluate sciatic nerve repair following peripheral nerve injury in varying levels of severity where nerve continuity is maintained (26, 27, 39) to complete disruption of axonal and surrounding connective tissue (44, 45). DTI has great potential in this regard as measurements are taken *in vivo*, precluding the need for large numbers of animals to be sacrificed at multiple time points. Due to limitations in data acquisition, these authors were able to reconstruct only small segments of the sciatic nerve and included spurious fibers. We present here robust tractography of the branches of the sciatic nerve from the dorsal root ganglion to their respective terminal muscle destinations, showing that accurate peripheral nerve tractography is achievable with appropriate acquisition parameters and post-processing techniques. Some of the above authors have used track length, normalized to a pre-surgical baseline, as a marker of nerve integrity. This can be problematic as the angle of nerve trajectory is a significant factor in fiber reconstruction and body position is not easily reproduced in repeated imaging sessions and between subjects. Nerve assessment via DTI metrics from a ROI, as we present here, may be a more robust method of evaluating nerve integrity.

The results of this study are consistent with previous experiments who reported similar observations of disrupted myelin and axon swelling in the sciatic nerves of pigs (19, 46) and rats (7) after treatment with FUS. These authors' observations are based on histology but are reinforced by our analysis of both histology and DTI metrics. This important validation of histological analysis by *in vivo* imaging obviates the need to sacrifice animals in order to assess the microstructural response to FUS treatment. DTI has been demonstrated previously as a useful adjunct to histological analysis (47, 48) and this study extends its application to MRgFUS.

We note that both Huisman et al. and Kaye et al. used large adult pigs (50–75 and 19–40 kg, respectively) with relatively large sciatic nerves (8–10 mm wide) (19, 46). Here we demonstrate the ability to image small animals (5–8 kg) and target small nerves (3–4 mm wide) with similar hardware. These smaller nerves reflect a similar size to potential human clinical MRgFUS targets such as the pudendal nerve (49) or posterior femoral cutaneous nerve (50), as noted by Huisman et al. (19).

Clinically, MRgFUS has potential as a non-invasive alternative to other ablation modalities, including radiofrequency ablation. Peripheral nerve ablation has been studied in a variety of cancerous (8, 9, 12, 13) and non-cancerous conditions (6, 10, 11). Ablation has also been used to treat tumors with close proximity to neural structures (14). For pain relief, a nerve conduction block may be indicated before ablative tract disruption is considered, as suggested by the findings of Foley et al. on FUS-related changes in nerve conduction (7). While conduction block can be successful in reducing pain, the effect can be limited in duration and nerve modulation or ablation may be further considered (51). Whether nerve conduction block or ablation is indicated, an *in vivo* imaging-based understanding of FUS-related nerve changes is important for characterizing treatments and predicting outcomes. This study demonstrates the utility of DWI to visualize and assess nerve changes in these treatments, thus bolstering the potential clinical utility of MRgFUS treatments.

We have demonstrated specificity on segmenting portions of the sciatic nerve based on vertebral origin and terminating muscle innervation (Supplementary Figure 1). The same seed ROIs at the nerve roots were used while disparate inclusion ROIs were placed at distal branches of the nerve. Similar techniques have been validated in the central nervous system for segmenting specific tract locations in thalamic nuclei by utilizing anatomically and functionally distinct cortical and subcortical regions in patients with essential tremor (25, 52). This demonstrates the potential to target specific portions of peripheral nerves in non-invasive treatments while avoiding branches of non-interest.

## Limitations

This is an acute study and thus we are not able to determine the longitudinal effect of single-exposure MRgFUS treatment including effects at the lesion site and distal portions of the treated nerve. Previous studies have suggested that Wallerian-type degeneration may cause disruption in nearby white matter and along the length of the lesioned tract (53). Further studies incorporating multiple imaging time points are needed to understand the evolution of MRgFUS lesions and microstructural changes at both the primary injury site and distal tract segments. This study also uses ablative treatments of peripheral nerves, whereas lower intensity, non-ablative sonication may be indicated, as in the case of nerve conduction blocks (7). Future longitudinal studies may thus also incorporate lower power MRgFUS treatments to assess sub-lesional nerve changes and the inflection point between ablative and non-ablative therapies.

Technical challenges limit three-dimensional tractograms from being visualized directly on the MRgFUS targeting software. As such, specific fiber positions must be inferred *in vivo* relative to nearby anatomical structures and with reference to pre-treatment planning images. There exists the potential for targeting errors due to operator bias and positioning reproducibility. This problem is not limited to MRgFUS treatments but with surgical interventions incorporating the visualization of the central and peripheral nervous systems in general. Stereotactic frames in cranial applications can aid target registration but such devices are not generally available for peripheral nerve treatments. Further development in adequate forms of repeatable limb positioning and immobilization is needed for coupling tractography directly with non-invasive surgical approaches to minimize the effect of movement and maximize targeting accuracy.

## Conclusions

This study demonstrates the potential of DWI and tractography for the *in vivo* targeting and assessment of changes following MRgFUS treatment of peripheral nerves. Significant changes in DTI metrics of FA, MD, AD, and RD were observed in the sciatic nerve following single exposure MRgFUS treatment. These DTI metric changes were correlated with sonication parameters. Robust visual reconstruction of the sciatic nerve via tractography was achieved. Diffusion imaging may thus be a valuable tool in optimizing peripheral nerve treatments with MRgFUS and evaluating the effects of treatment.

## Data Availability Statement

The raw data supporting the conclusions of this article will be made available by the authors, without undue reservation.

## Ethics Statement

The animal study was reviewed and approved by Animal Care Committee and Laboratory Animal Services at the Hospital for Sick Children, Toronto, Ontario, Canada.

## Author Contributions

MW, JZ, AW, KP, JD, and MH participated in the experimental design. Data was collected by MW, JZ, AW, and KP. The manuscript was written by MW. Manuscript critique and interpretation was contributed by JZ, AW, KP, LN, DA, JD, and MH. All authors contributed to this work and contributed to the analysis.

## Conflict of Interest

The authors declare that the research was conducted in the absence of any commercial or financial relationships that could be construed as a potential conflict of interest.

## Supplementary Material

Supplementary Figure 1 CSD tractography of the sciatic nerve segmented by fiber bundle vertebral origin and distal termination (individual tracts separated by color). Full reconstructed tracts visible from **(A)** axial, **(B)** sagittal, and **(C)** coronal view. **(D)** Axial views showing only tract segments within the visible slice centered on the MRgFUS lesion. **(E)** Lesion zones I (red) and II (blue) are highlighted. CSD, constrained spherical deconvolution; MRgFUS, magnetic resonance-guided focused ultrasound.
