# Supplementary figures and images for: Peripheral Nerve Focused Ultrasound Lesioning—Visualization and Assessment Using Diffusion Weighted Imaging
Source: Front Neurol. 2021 Jul 9;12:673060. doi: 10.3389/fneur.2021.673060 (PMC8299784; doi:10.3389/fneur.2021.673060)

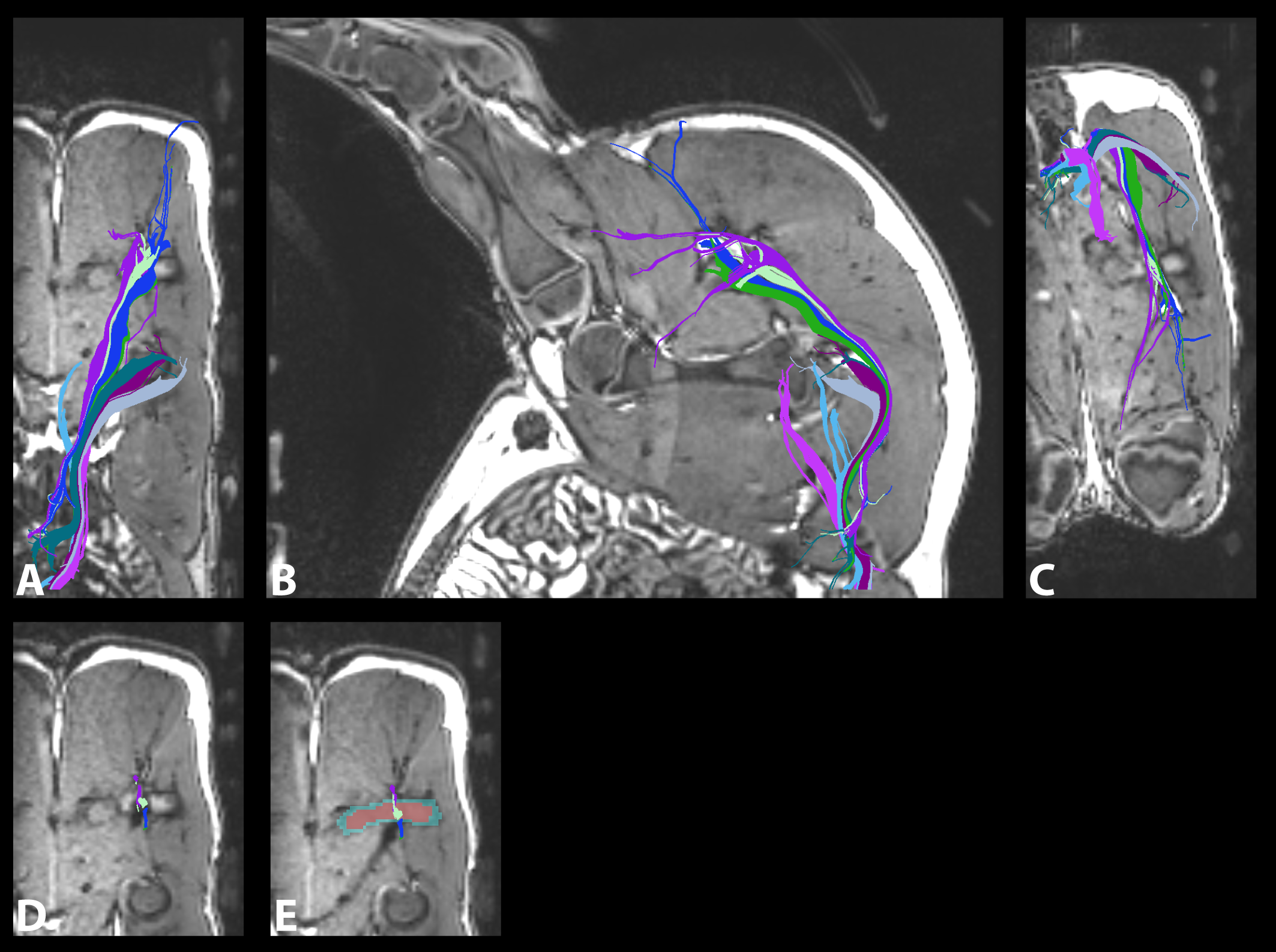

Supplement: Supplementary Figure 1 — CSD tractography of the sciatic nerve segmented by fiber bundle vertebral origin and distal termination (individual tracts separated by color). Full reconstructed tracts visible from (A) axial, (B) sagittal, and (C) coronal view. (D) Axial views showing only tract segments within the visible slice centered on the MRgFUS lesion. (E) Lesion zones I (red) and II (blue) are highlighted. CSD, constrained spherical deconvolution; MRgFUS, magnetic resonance-guided focused ultrasound. [file Image_1.TIF]
